# In Vitro Study of Vitamin D Effects on Immune, Endothelial, and Vascular Smooth Muscle Cells in Chronic Kidney Disease

**DOI:** 10.3390/ijms26093967

**Published:** 2025-04-23

**Authors:** Kajal Kamboj, Vivek Kumar, Ashok Kumar Yadav

**Affiliations:** 1 Departments of Nephrology, Postgraduate Institute of Medical Education and Research, Chandigarh 160012, India; nadhakajal@gmail.com (K.K.); enigma165@yahoo.co.in (V.K.); 2 Experimental Medicine and Biotechnology, Postgraduate Institute of Medical Education and Research, Chandigarh 160012, India

**Keywords:** cholecalciferol, immune cells, human aortic endothelial cells, human aortic smooth muscle cells, PBMCs, vascular function

## Abstract

Vitamin D has been shown to improve immunity as well as vascular function. We investigated the effect of cholecalciferol on T-cell phenotype in cultured peripheral blood mononuclear cells (PBMCs) from twenty vitamin D-deficient, non-diabetic chronic kidney disease (CKD) subjects. We also studied vitamin D effects on endothelial and vascular function markers in human aortic endothelial cells (HAECs) and in human aortic smooth muscle cells (HASMCs), respectively. We studied endothelial nitric oxide synthase (eNOS), mitogen-activated protein kinase 38 (p38 Map kinase), protein kinase B (Akt), and nicotinamide adenine dinucleotide phosphate oxidase (NADPH oxidase) in HAECs and α-smooth muscle actin (α-SMA), smooth muscle calponin (SM-Calponin), smooth muscle myosin heavy chain (SM-MHC), and calcium-sensing receptor (CaSR) in HASMCs. Vitamin D receptors (VDRs) and CYP27B1 were studied in both cell types. In cultured PBMCs isolated from CKD subjects, the percentage of T helper 1(TH1) cells significantly decreased while that of T helper 2 (TH2) cells increased after cholecalciferol treatment. No significant change in intracellular and surface markers of T helper 17 (TH17) and T regulatory (Treg) cells was observed. In vitro treatment of HASMCs and HAECs with cholecalciferol led to significant and favorable alterations in mRNA expression of markers of vascular smooth muscle cells, i.e., *α-SMA*, *SM-Calponin*, and *SM-MHC*. Regarding endothelial cell markers, mRNA encoding *eNOS*, *p38 Map kinase*, *protein kinase B (Akt)*, *NADPH oxidase*, *VDR*, and *CYP27B1* were also significantly changed. Finally, the expression levels of the following proteins were notably altered: NADPH oxidase and protein kinase B (Akt) (in HAECs); SM-MHC and SM-Calponin (in HASMCs). In vitro treatment of PBMCs with cholecalciferol led to a favorable change in T-cell population, decreasing TH1 and increasing TH2 cell percentage, along with beneficial alterations in mRNA expression of HASMCs and HAECs’ cell markers. This study provides evidence that cholecalciferol can influence immune and vascular function in CKD.

## 1. Introduction

Chronic kidney disease (CKD) is a long-term condition with a decline in renal function assessed by glomerular filtration rate (<60 mL/min/1.73 m^2^) or kidney damage (proteinuria > 500 g/day) caused by various conditions such as diabetes and hypertension, leading to end-stage kidney disease (ESKD) or kidney failure. Patients with CKD experience higher rates of morbidity (60%) and mortality (40%) compared to individuals with normal kidney function, particularly because of cardiovascular complications in CKD in addition to a higher risk of developing ESKD [1,2]. Compared to the general population, CKD patients have significant immune dysregulation, an increased incidence of malignancy, higher susceptibility to infections, and poor response to vaccination [3,4,5,6]. The decline in kidney function affects the innate and adaptive immune system cells differently regarding the cell’s number and activation status. T-cell proliferation in vitro is reduced in a uremic environment [7,8,9,10]. In addition, CKD patients present with lower circulatory counts of T-lymphocytes (CD4^+^, CD8^+^) and B lymphocytes [11].

In CKD, factors like immune dysregulation, oxidative stress, uremic toxins, and hypertension can trigger inflammation and, hence, functional deterioration of the endothelial cells (ECs), which consequently elevates the risk of cardiovascular complications. Endothelial dysfunction in CKD patients is indicative of multifaceted endothelial injury combined with impaired endothelial repair and regeneration. A hallmark of endothelial dysfunction in CKD is the diminished bioavailability of a gaseous molecule, nitric oxide (NO), known for its anti-inflammatory, vasorelaxant, and antithrombotic properties [12]. Recent research revealed the molecular mechanisms underlying endothelial dysfunction in CKD [13,14].

In addition, there is evidence of the link between low vitamin D levels and increased mortality and morbidity in CKD [15]. Hence, supplementation of vitamin D may improve survival rate in CKD patients [16,17,18]. Numerous reports show how patients with CKD or ESKD responded to the vitamin D supplementation, and many studied the involvement of biomolecules from cellular pathways linked to vitamin receptor (VDR). Experimental studies on humans and animals have shown the role of vitamin D in cell proliferation, differentiation, and apoptosis [19,20]. Administering a high dose of vitamin D to healthy volunteers substantially elevated the Treg population among the peripheral blood mononuclear cells (PBMCs) [21]. In another study, calcitriol treatment in renal transplant recipients caused an increase in Treg population [22]. Calcitriol is known as 1,25-dihydroxycholecalciferol [1,25 (OH)_2_D_3_] and represents an active form of vitamin D. The supplementation of the active form of vitamin D, i.e., 1,25 (OH)_2_D_3_, successfully promoted TH2 cell differentiation in hemodialysis patients [23]. In vitro and in vivo studies have also demonstrated the effects of vitamin D on the cardiovascular system, such as inhibiting cardiomyocyte proliferation, regulating vascular smooth muscle cell proliferation, promoting endothelial growth factor expression, and regulating both the renin–angiotensin–aldosterone system and natriuretic peptide secretion [24]. Previously, we have shown how cholecalciferol improved endothelial function in CKD patients by measuring the flow-mediated dilatation, pulse wave velocity, and circulating endothelial and inflammatory markers [25,26]. From a pathological perspective in CKD patients, it became evident that cardiovascular complications, infections, immune dysfunction, and the inflammatory response are all intertwined and can be affected by vitamin D treatment. However, studies investigating the in vitro and ex vivo effects of vitamin D on vascular and smooth cell function and immune function are limited. In the current study, we investigated the in vitro effect of cholecalciferol on T-cell phenotypes in PBMCs originating from CKD subjects. Moreover, we report how vitamin D affected endothelial and vascular cell function markers HAECs and HASMCs.

## 2. Results

### 2.1. T-Cell Phenotype in CKD Subjects Is Altered upon Vitamin D Supplementation

**Figure 1:**
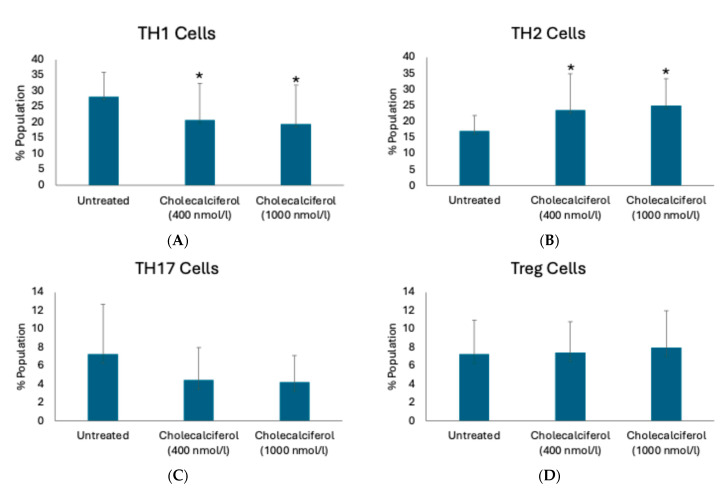
T-cell phenotypes alter after the cholecalciferol treatment at two doses, as analyzed by flow cytometry. (**A**) TH1 CD3^+^CD4^+^IFNγ^+^. (**B**) TH2 CD3^+^CD4^+^STAT6^+^. (**C**) TH17 CD3^+^CD4^+^IL17A^+^. (**D**) Treg CD3^+^CD4^+^CD25^+^CD127^low^FOXP3^+^ cell population. * *p* < 0.05; *p*-values were obtained using the Wilcoxon signed-rank test, and T-cell phenotype was analyzed in PBMCs taken from twenty CKD subjects.

We phenotyped T-cells in two separate assays: (1) T-cells from the PBMCs, which were first isolated from twenty CKD subjects and then supplemented with cholecalciferol (ex vivo experiment), and (2) T-cells originating from the whole blood taken from the same CKD patients. In the latter case, T-cells were isolated at the baseline (before the cholecalciferol supplementation) and post-supplementation. As shown in Table 1 and Figure 1, the percentage of TH1 cells with CD3^+^CD4^+^IFNγ^+^ phenotype significantly decreased after treatment with cholecalciferol at both tested concentrations (400 and 1000 nmol/L) compared to untreated cells. In contrast, the percentage of TH1 cells with CD3^+^CD4^+^Tbet^+^ phenotype or those with CD3^+^CD4^+^CXCR3^+^ phenotype did not change.

Regarding TH2 cells, the percentage of TH2 cells with CD3^+^CD4^+^STAT6^+^ phenotype and those with CD3^+^CD4^+^CCR4^+^CCR6^−^ phenotype significantly increased after cholecalciferol treatment at both tested concentrations compared to untreated. In contrast, the other two examined TH2 subsets that carry either IL4 or GATA did not change (Table 1, Figure 1). Of all the tested TH17 cell subsets, only those carrying CCR4^+^ and CCR6^+^ cell markers significantly altered upon the treatment with cholecalciferol at a 400 nmol/L dose. The alterations in the ratios of Treg cell subsets could not be observed in the studied conditions (Table 1, Figure 1).

Figure 1 shows T helper cell subpopulations analyzed in PBMCs isolated from the CKD subjects and treated in vitro without (untreated) or with cholecalciferol at two doses: 400 nmol/L and 1000 nmol/L. The data depicted in Figure 1 are similar to data obtained from studying T-cell phenotypes in CKD subjects who were given vitamin D supplementation, as shown in Figure S1. Individual-level data of T-cell subsets in CKD (pre- and post-vitamin D supplementation) and cultured PBMCs (untreated and treated with 400 and 1000 nmol/L cholecalciferol), as represented in Figure S2, show a similar trend.

### 2.2. Expression of mRNA Encoding Vascular Smooth Muscle Cell Markers Alters upon the Cholecalciferol Treatment

The expression of mRNAs that encode HASMC markers *α-SMA*, *SM-MHC*, and *SM-Calponin*, alongside mRNAs for *VDR* and *CYP27B1*, was examined and compared in the untreated control cells, treated control cells (ethanol treatment), and cells treated with cholecalciferol, as shown in Figure S3. The expression of four mRNAs rose significantly after the supplementation, the most pronounced being mRNA encoding *SM-MHC*, which was almost eleven times higher in the cholecalciferol-treated HASMCs than in the untreated controls. Since no significantly different expression of these five mRNAs could be observed between the untreated and ethanol-treated control cells, we omitted the former from further assays and used only the latter in comparisons with the cholecalciferol-treated cells (Figure S3).

**Figure 2:**
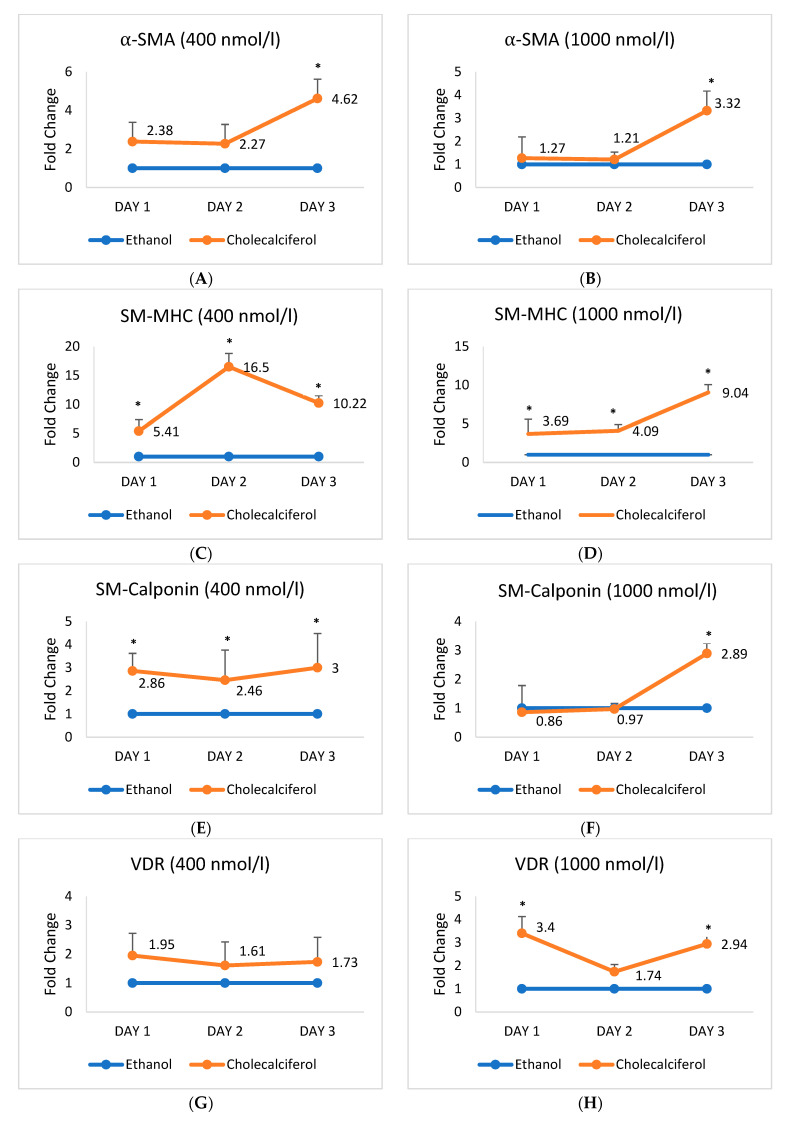

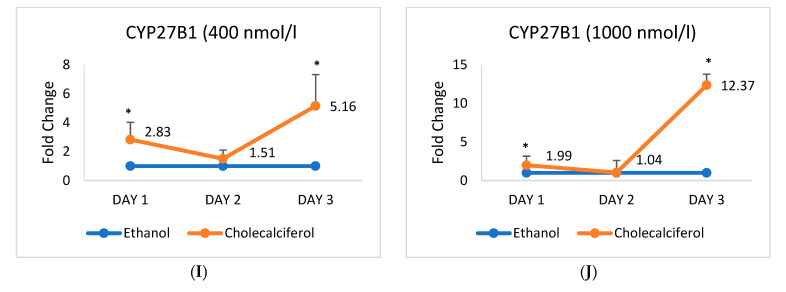
mRNA expression of markers of HASMCs, *VDR*, and *CYP27B1* on days 1, 2, and 3 after cholecalciferol treatment. Ethanol (400 nmol/L and 1000 nmol/L) was used as a treated control. mRNA expression (**A**) *α-SMA* at 400 nmol/L (**B**) *α-SMA* at 1000 nmol/L (**C**) *SM-MHC* at 400 nmol/L (**D**) *SM-MHC* at 1000 nmol/L (**E**) *SM-Calponin* at 400 nmol/L (**F**) *SM-Calponin* at 1000 nmol/L (**G**) *VDR* at 400 nmol/L (**H**) *VDR* at 400 nmol/L (**I**) *CYP27B1* at 400 nmol/L (**J**) *CYP27B1* at 1000 nmol/L cholecalciferol. * *p* < 0.05 was significant. *p*-values were obtained using the Wilcoxon signed-rank test. α-SMA: α-smooth muscle actin; SM-MHC: smooth muscle myosin heavy chain; SM-Calponin: smooth muscle calponin; VDR: vitamin D receptor; CYP27B1: cytochrome P450, family 27, subfamily B, member 1.

HASMCs were either exposed to ethanol at two doses (400 and 1000 nmol/L) or to cholecalciferol at the same doses (400 and 1000 nmol/L), followed by measurements of mRNA encoding HASMC cell markers *α-SMA*, *SM-MHC*, *SM-Calponin*, *VDR*, and *CYP27B1* on days 1, 2, and 3 after the treatment. The mRNA ratios were expressed as fold change and shown in Figure 2.

One day after the cholecalciferol treatment of HASMCs, mRNAs encoding *SM-MHC* and *CYP27B1* were significantly increased at both applied doses; mRNAs for SM-Calponin at 400 nmol/L and *VDR* at 1000 nmol/L were also significantly increased. On day 2, mRNAs for SM-MHC at both doses were increased. mRNA for *SM-Calponin* reacted only to 1000 nmol/L of cholecalciferol. However, on day 3, the expression of all five studied mRNAs was augmented at 400 and 1000 nmol/L, the only exception being mRNA for *VDR* at 400 nmol/L. *SM-MHC* mRNA experienced the biggest change upon the cholecalciferol treatment of HASMCs, over nine-fold compared to the controls.

### 2.3. Expression of mRNA Encoding Endothelial Cell Markers Alters upon the Cholecalciferol Treatment

**Figure 3:**
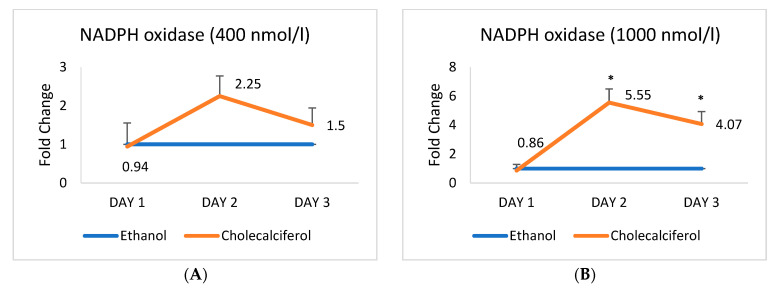

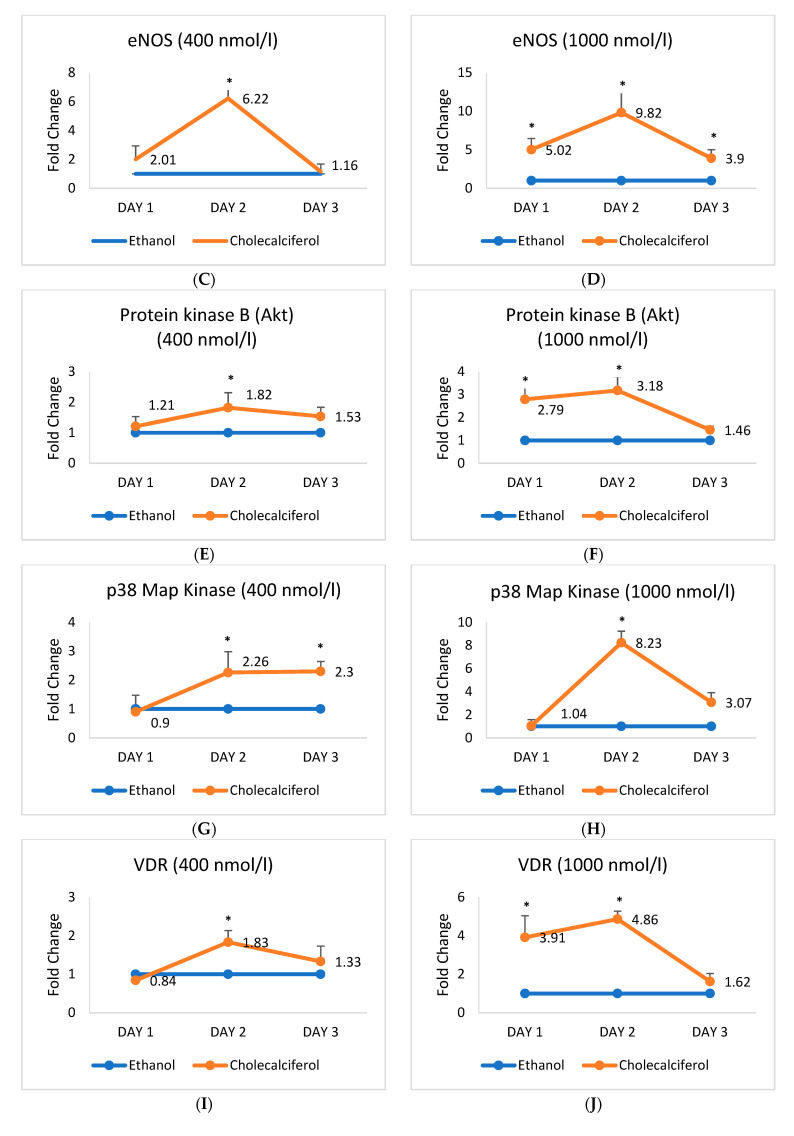

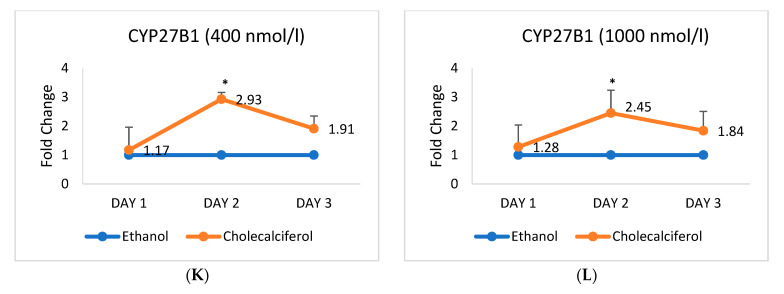
mRNA expression of markers of HAEC, VDR, and CYP27B1 on days 1, 2, and 3 after cholecalciferol treatment. Ethanol (400 nmol/L and 1000 nmol/L) was used as a treated control. mRNA expression (**A**) *NADPH oxidase* at 400 nmol/L (**B**) *NADPH oxidase* at 1000 nmol/L (**C**) *eNOS* at 400 nmol/L (**D**) *eNOS* at 1000 nmol/L (**E**) *Protein kinase B (Akt)* at 400 nmol/L (**F**) *Protein kinase B (Akt)* at 1000 nmol/L (**G**) *p38 Map Kinase* at 400 nmol/L (**H**) *p38 Map Kinase* at 400 nmol/L (**I**) *VDR* at 400 nmol/L at 400 nmol/L (**J**) *VDR* at 400 nmol/L at 1000 nmol/L (**K**) *CYP27B1* at 400 nmol/L (**L**) *CYP27B1* at 1000 nmol/L (cholecalciferol. * *p* < 0.05 was taken significant. *p*-values were obtained using the Wilcoxon signed-rank test. NADPH oxidase: nicotinamide adenine dinucleotide phosphate oxidase; eNOS: endothelial nitric oxide synthase; protein kinase B (Akt): protein kinase B serine/threonine kinase; p38 Map kinase: mitogen-activated protein kinase 38; VDR: vitamin D receptor; CYP27B1: cytochrome P450, family 27, subfamily B, member 1.

HAECs were either exposed to ethanol at two doses (400 and 1000 nmol/L) or to cholecalciferol at the same doses (400 and 1000 nmol/L) for 24 h, followed by measurements of mRNAs encoding HAEC cell markers: *NADPH oxidase*, *eNOS*, *protein kinase B (Akt)*, and *p38 MAPK*, along with mRNAs for *VDR* and *CYP27B1*, on days 1, 2, and 3 after the treatment. The mRNA ratios were expressed as fold change and shown in Figure 3.

Significant changes in mRNA expression were observed in most cases after the cholecalciferol treatment of HAECs. The most profound changes were those in the expression of mRNA encoding *eNOS* on day 2 (almost a ten-fold increase in mRNA) at 1000 nmol/L cholecalciferol. Interestingly, mRNA for *NADPH oxidase* changed only when the cells were treated with 1000 nmol/L of cholecalciferol (Figure 3). The latter dose generally triggered greater changes in the expression of examined mRNAs, which also peaked on day 2 in most cases.

### 2.4. Expression of Protein Markers of HASMCs and HAECs Alters upon the Cholecalciferol Treatment

**Figure 4:**
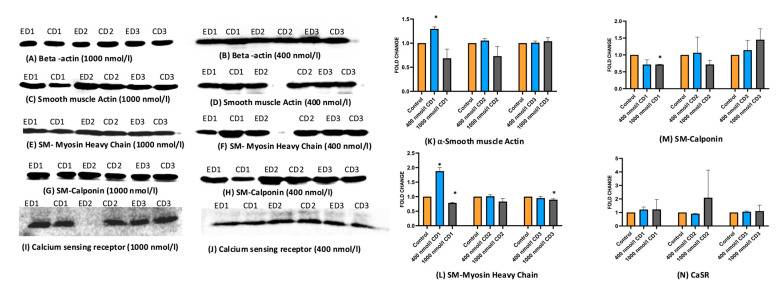
Protein expression of markers of HASMCs on days 1, 2, and 3 after cholecalciferol treatment. (**A**–**J**): Western blot bands of respective protein markers. (**K**–**N**): Bar graph showing the fold change in expression of the HAECs protein markers Beta-actin was used as a housekeeping gene. * *p* < 0.05 was significant. *p*-values were obtained using the Wilcoxon signed-rank test. α-SMA: α-smooth muscle actin; SM-MHC: smooth muscle myosin heavy chain; SM-Calponin: smooth muscle calponin; CaSR: calcium-sensing receptor; ED1: ethanol day 1; CD1: cholecalciferol day 1; ED2: ethanol day 2; CD2: cholecalciferol day 2; ED3: ethanol day 3; CD3: cholecalciferol day 3.

**Figure 5:**
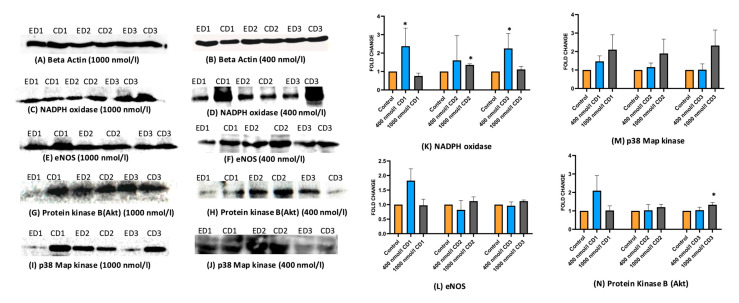
Protein expression of markers of HAECs analyzed by Western blot on days 1, 2, and 3 after cholecalciferol treatment. (**A**–**J**): Western blot bands of respective protein markers. (**K**–**N**): Bar graph showing the fold change in expression of the HAECs protein markers. Beta-actin was used as a housekeeping gene. * *p* < 0.05 was significant, and *p*-values were obtained using the Wilcoxon signed-rank test. NADPH oxidase: nicotinamide adenine dinucleotide phosphate oxidase; eNOS: endothelial nitric oxide synthase; p38 Map kinase: mitogen-activated protein kinase 38; protein kinase B (Akt): protein kinase B serine/threonine kinase; ED1: ethanol day 1; CD1: cholecalciferol day 1; ED2: ethanol day 2; CD2: cholecalciferol day 2; ED3: ethanol day 3; CD3: cholecalciferol day 3.

HASMCs and HAECs were exposed to two different doses of cholecalciferol: 400 and 1000 nmol/L, and further incubated 1, 2, or 3 days in cell medium. Total proteins were then extracted from these cell lysates and subjected to Western blotting to assess the levels of the following proteins: α-SMA, SM-MHC, SM-Calponin, calcium-sensing receptor (CaSR) in HASMCs, NADPH oxidase, eNOS, protein kinase B (Akt), and p38 Map kinase in HAECs. Figure 4 and Figure 5 depict the protein bands resolved by electrophoresis and visualized by Western blotting. The bands were subjected to densitometry, and obtained protein levels were compared between the groups: control, treated at 400 nmol/L, and treated at 1000 nmol/L cholecalciferol. The significant changes in the protein expression compared to controls were observed in the following cases: α-SMA and SM-MHC increased after the exposition to 400 nmol/L cholecalciferol after one day; SM-MHC and SM-Calponin increased after the exposition to 1000 nmol/L cholecalciferol after one day. No markers appeared to be different on day 2. Finally, on day 3, only SM-MHC expression changed, and at 1000 nmol/L. No change in CaSR expression was observed at either concentration (Figure 4).

As shown in Figure 5, the treatment of HAECs with 400 nmol/L of cholecalciferol significantly increased the expression of NADPH oxidase on days 1 and 3, whereas the treatment with 1000 nmol/L of cholecalciferol increased the levels of this enzyme on day 2. No significant changes in the expression of p38 Map kinase and eNOS in HAECs treated with cholecalciferol were detected. However, the levels of Akt kinase increased on day 3 after the cells were treated with 1000 nmol/L cholecalciferol.

Nitrite and nitrate concentrations in the supernatants from HAECs treated with cholecalciferol at two doses (400 and 1000 nmol/L) did not change significantly compared to the non-treated controls on days 1, 2, and 3 (Figure S4).

## 3. Discussion

In the study reported herein, we present the effects of vitamin D supplementation given to twenty CKD patients on their T-cell phenotype. T-cells originated from the PBMC fraction isolated from the whole blood of the patients, pre- and post-supplementation. We have also phenotyped different populations of T helper cells from the PBMCs in culture, which were isolated from the same patients. Finally, we describe the effects of in vitro incubation of human aortic smooth muscle and endothelial cells (HASMCs and HAECs, respectively) with cholecalciferol, focusing on the cell markers of immunity and vascular functions.

T-cell phenotypes were altered in the blood of CKD patients that were given cholecalciferol. Moreover, the T-cell phenotype studied ex vivo, after the PBMCs were exposed to cholecalciferol at two different doses, showed similar changes. These changes can be summed up as a significant drop in a TH1 cell subpopulation accompanied by a rise in TH2 cell subpopulations. However, not all TH cell subpopulations responded to the cholecalciferol supplementation or cholecalciferol treatment ex vivo. Among TH1, only those cells bearing interferon gamma, i.e., CD3^+^CD4^+^IFNγ^+^, reacted to the exposition to cholecalciferol. Among TH2, the reactive T-cell subpopulations were those bearing STAT6 (CD3^+^CD4^+^STAT6^+^) or those bearing CCR4^+^ but not CCR6^−^ (CD3^+^CD4^+^CCR4^+^CCR6^−^). The effects of the ex vivo supplementation were dose-dependent, where 1000 nmol/L caused greater change in T-cell phenotypes than 400 nmol/L of cholecalciferol.

Interestingly, TH17 cell subpopulations proved to be more resistant to the supplementation. In this case, only the change in percentage of CD3^+^CD4^+^CCR4^+^CCR6^+^ was found to be significant, and only at 400 nmol/L of cholecalciferol. Finally, we could not observe any change in Treg cell ratios upon the supplementation.

The in vitro exposure of HASMCs and HAECs to cholecalciferol resulted in altered expression of specific mRNAs and respective proteins (those encoded by these mRNAs). These proteins are functional markers of smooth muscle cells and endothelial cells. Moreover, mRNA transcribed from the genes responsive to vitamin D was also altered.

The abnormalities in T-cell phenotype and favorable effect of vitamin D on T-cells in CKD have been observed [27,28]. In our study, analysis of PBMCs from CKD subjects showed that cholecalciferol significantly decreased T-cell subsets with IFNγ and increased T-cell subsets with STAT6, but no change was observed in T-cells carrying IL-4. It has been reported that the IL-4 receptor, upon being activated by binding to IL-4, triggers the STAT6 activation, favoring the TH2 differentiation pathway [29]. During an in vitro study of CD4^+^ T-cells, 1,25(OH)_2_D_3_ showed inhibitory effects on IFN-γ and IL-4 in the early stage of polarization. Furthermore, the capability of 1,25(OH)_2_D_3_ to downregulate IL-4 levels under polarizing conditions while boosting levels of this cytokine under nonpolarizing conditions indicates a distinct regulation of IL-4 by 1,25(OH)_2_D_3_. This modulation might rely on the cytokine milieu of IFN-γ and IL-4 [30].

Correale et al. reported a decrease in the percentage of CD4^+^ T-cells carrying IL-17 (TH17 cells) in PBMCs following in vitro treatment with 1,25(OH)_2_D_3_ in humans [31]. In our study, cholecalciferol treatment did not significantly change the TH17 cells (CD4^+^IL-17^+^) percentage, although a decreasing trend was observed. However, a significant change occurred in TH17 cells with the CD3^+^CD4^+^CCR4^+^CCR6^+^ phenotype in our study. Correale et al. also reported an increased percentage of FOXP3^+^CD4^+^ T-cells, while we did not see an altered percentage of Tregs carrying FOXP3. Other studies have also reported no effect of high or low doses of vitamin D on Treg cell population in diabetes and healthy individuals [32,33]. The insignificant effect of cholecalciferol on Treg cells might be due to inadequate response of Treg cells to activating signals in CKD. Whether alterations in the proportion of Tregs in peripheral blood cells correspond to potential changes in Treg cells at the tissue level is still unclear. The effect of cholecalciferol on T-cell phenotype remained similar in ex vivo treatment as well as pre- and post-cholecalciferol supplementation in CKD patients. The variable effect of vitamin D on T-cell phenotype across various studies might be attributed to several factors, including the state of T-cell activation, CYP27B1 activity, which converts inactive vitamin D to active vitamin D, and levels of 25(OH)D_3_ or 1,25(OH)_2_D_3_. 

In healthy arteries, vascular smooth muscle cells highly express α-SMA along with other structural and actin-binding proteins, collectively defining the contractile phenotype of vascular smooth muscle cells. However, in disease conditions following arterial injury, α-SMA expression and other structural protein expressions decrease, leading vascular smooth muscle cells (VSMCs) to adopt a proliferative and promigratory phenotype [34]. As biological actions of activated vitamin D are mediated by its receptor VDR and CYP27B1, we also studied these markers. Our study showed that varying doses of cholecalciferol demonstrated a beneficial alteration in the mRNA expression of these markers of HASMC and HAEC function in vitro. mRNA expression of vascular smooth muscle cell function markers, i.e., *α-SMA*, *SM-MHC*, *SM-Calponin,* and vitamin D-responsive genes, i.e., *VDR* and *CYP27B1*, significantly increased after cholecalciferol treatment. However, the protein expression was inconsistent, as some of the protein had significantly increased expression (SM-MHC and SM-Calponin), while for some (α-SMA), no changes were observed after cholecalciferol treatment. Additionally, we observed an increased expression of VDR and CYP27B1, suggesting a potential role for cholecalciferol being converted to its active form and interacting with endothelial cells. Vitamin D induced time- and dose-dependent increases in VDR expression as well as relaxation by phosphorylation of myosin phosphatase target subunit 1^Ser507^ after treatment of placental VSMCs with 1,25 (OH)_2_D_3_ [35].

Cholecalciferol effectively increased the mRNA expression of endothelial cell function markers, i.e., *NADPH oxidase*, *eNOS*, *protein kinase B (Akt)*, and *p38 Map Kinase*, as well as *VDR* and *CYP27B1* in HAECs. Protein expression of NADPH oxidase and protein kinase B (Akt) increased after cholecalciferol treatment, while no changes were observed in other proteins. Increased expression of *CYP27B1* was observed in this study, which suggests the endothelial synthesis of 1,25(OH)_2_D_3_. Our finding of increased *VDR* expression in HAECs suggests that these cells may have a vitamin D biosynthesis auto-regulatory system, as shown in a study by Ma et al. [36]. Vitamin D has also been reported to stimulate NO production in cultured human umbilical vein endothelial cells (HUVECs) by activating eNOS, and it was mediated by VDR, which causes a significant upregulation in the phosphorylation levels of intracellular kinases, including p38, protein kinase B (Akt), and extracellular signal-regulated kinases, leading to eNOS activation [37]. However, in our study we did not find any change in concentrations of nitrite/nitrate in cell supernatant of cells treated with cholecalciferol by Griess’s method, which might be due to time-dependent decrease in NO concentrations as reported by Molinari et al. [37], which showed optimal NO production at 1 min and decreased progressively after 1 min [37]. In one other study on HUVECs, it was shown that endothelial β_2_-adrenoceptor-mediated NOS-3 (eNOS) activation was also mediated through phosphorylation of NOS-3, through both the protein kinase A (PKA) and Akt [38]. Similar to our results, vitamin D has been reported to affect the proliferation of endothelial cells [39].

It is important to note that the detection of protein expression of these markers in HASMCs and HAECs varies depending on the protein extraction methods and the variability in types of primary antibody used. Consequently, researchers have encountered difficulties in detecting these proteins [40]. Furthermore, there are discrepancies in studies regarding the form and dosage of vitamin D used, cell line used, and time period of treatment, hence leading to varying outcomes. Overall, we have shown that vitamin D affects functional protein markers in HASMC and HAEC cells, which can have consequences on the whole vascular endothelial system. Thus, it can be inferred that increased expression of α-SMA, SM-MHC, and SM-Calponin results in decreased proliferation and hence decreased calcification in VSMCs [41,42]. Vitamin D deficiency lowers the levels of calcification inhibitors and heightens the inflammatory response, contributing to increased vascular calcification [43]. An in vitro study of VSMCs exposed to serum obtained from uremic rats also showed increased 1α-hydroxylase expression, and these increases were parallel to an increase in vascular calcification [44]. Consequently, the tight regulation of vitamin D levels is crucial for vascular function, and our dosage treatments showed a positive response.

Our study clearly demonstrates that administering cholecalciferol affects the T-cell phenotype in vitro as well as in CKD patients. Cholecalciferol also increased the mRNA expression of *NADPH oxidase*, *eNOS*, *p38*, and *Akt*. These molecules are known to be involved in the intracellular signaling pathways that lead to NO production. NO induces the expression of contractile proteins in HASMC. Indeed, ECs regulate the quality and characteristics of the underlying VSMCs by releasing various relaxing and contracting factors as well as through direct interactions with these relaxing and contracting factors, as shown by increased mRNA expression of *α-SMA*, *SM-MHC*, and *SM-Calponin* in our study.

The data from our study indicate that cholecalciferol is effective in favorably influencing the immune as well as vascular function and support the finding of our previous study reporting the improvement of endothelial function in CKD after vitamin D supplementation [25,26].

In conclusion, cholecalciferol supplementation was effective in improving the immune and vascular function, as shown by beneficial changes in TH1 and TH2 cell phenotypes and markers of endothelial and smooth muscle cell function. Further studies are needed to explore the detailed mechanistic pathways and/or factors that are regulated by vitamin D and affecting the process of vascular dysfunction in CKD.

## 4. Materials and Methods 

### 4.1. Study Design

This in vitro study analyzed the effect of cholecalciferol treatment in vascular smooth muscle cells and endothelial cells. In addition, we examined T-cell phenotypes. HAECs, HASMCs, and PBMCs from CKD subjects were used for analysis.

### 4.2. Study Population for T-Cell Phenotyping

T-cell phenotyping was carried out by in vitro assay and ex vivo assay (Supplementary Material). T-cell phenotyping encompassed determining the percentage of T helper cell populations: TH1, TH2, and TH17, along with T regulatory (Treg) cells. For the ex vivo effect of cholecalciferol on change in expression of T-cell phenotypes, whole blood was taken from CKD subjects at baseline (before cholecalciferol supplementation), PBMCs were then isolated and treated with cholecalciferol. Twenty non-diabetic CKD patients (ages range 18–75 years) with an estimated glomerular filtration rate (eGFR) of 15–60 mL/min/1.73 m^2^ and serum 25 (OH) D levels <20 ng/mL were enrolled from an ongoing clinical trial of vitamin D in CKD at the Postgraduate Institute of Medical Education and Research (PGIMER), Chandigarh, India. GFR was estimated by the Chronic Kidney Disease Epidemiology Collaboration in 2009 (CKD-EPIcr_2009_) [45]. This study’s protocol was approved by the Institute’s Ethics Committee (IEC) (No: NK/6731/PhD/930 dated 1 December 2020). This study was registered in the Clinical Trial Registry of India (CTRI/2019/10/021494). Detailed inclusion and exclusion criteria are provided in Supplementary Materials. In addition to cultured PBMCs, T cells from these patients were phenotyped pre- and post-vitamin D supplementation. Briefly, the CKD patients were given two oral doses of 300,000 IU of cholecalciferol eight weeks apart (at baseline and at eight weeks), and subjects were followed up for 16 weeks.

### 4.3. PBMC Culture

PBMCs from the whole blood of study subjects were extracted by Ficol-histopaque. The PBMC pellet was then resuspended in cell culture media consisting of RPMI-1640 (Lonza, Walkersville, MD, USA, Cat. No. 12-702F) plus 10% FBS (Sigma-Aldrich, St. Louis, MO, USA, Cat. No. F7524) and 2% antibiotic–antimycotic solution (Sigma-Aldrich, St. Louis, MO, USA, Cat. No. A5955) containing 10,000 units penicillin, 10 mg streptomycin, and 25 μg amphotericin B per ml. Cells were revived overnight in a 5% CO_2_ incubator at 37 °C. Trypan blue stain (Sigma-Aldrich, St. Louis, MO, USA, Cat. No. T8154) was used for the measurement of cell viability. Cells were seeded in a 6-well plate (approximately 1 × 10^6^ cells in each well) and incubated with a solution of cholecalciferol (Sigma-Aldrich, St. Louis, MO, USA, Cat. No. C1357) at three different concentrations: 0 (control), 400, and 1000 nmol/L cholecalciferol. After 24 h of treatment, the cells were pelleted down, resuspended in phosphate-buffered saline, and processed further for T-cell subset analysis by flow cytometry.

#### 4.3.1. Flow Cytometry Analysis

T-cell subsets were analyzed in (a) cultured PBMCs after cholecalciferol treatment and (b) freshly isolated PBMCs from CKD subjects pre- and post-vitamin D supplementation. The control PBMCs were analyzed in parallel. Multicolor flow cytometry was used to discover different T-cell populations. The T-cell subset panel was prepared with primary antibodies against various surface markers, intracellular markers, and transcription factors in conjugation with different fluorescent markers (Becton Dickinson Biosciences, Franklin Lakes, NJ, USA) (Table S1). Compensation control (BD Biosciences, USA) was used to correct the fluorescence of various CD markers in the panel. The Flow Minus One (FMO) gating strategy was used to analyze the various subpopulations in T-cell panels. The acquisition of cells was performed on BD-FACS Aria, and the percentage of cells was analyzed using FACS-Diva software version 9.4 (Becton Dickinson Biosciences, Franklin Lakes, NJ, USA).

#### 4.3.2. Endothelial and Vascular Cell Culture

HASMCs (Human Primary Aortic Smooth Muscle Cells; normal, Cat. No. CC-2571) and HAECs (Human Primary Aortic Endothelial Cells; normal, Cat. No. CC-2535) were obtained from Lonza Walkersville, MD, USA. Smooth Muscle Cell Growth Medium-2 Bulletkit™ (SmGM™-2, Cat. No. CC-3182) containing smooth muscle cell basal media and Endothelial Cell Growth Medium Bulletkit™ (EGM™, Cat. No. CC-3124) and all the components/supplements of complete culture medium were used as per manufacturer’s instructions. Antimicrobials (Sigma-Aldrich, St. Louis, MO, USA, Cat. No. A5955) were added to the complete growth media as recommended by the manufacturer. Both cell types were incubated in 95% humidified air and 5% CO_2_ at 37 °C. When the cells attained approximately 90% confluency, they were passaged using trypsin-EDTA (Sigma-Aldrich, St. Louis, MO, USA, Cat. No. T3924). Cells cultured for 4–6 passages were invariably used.

Briefly, each T75 flask was seeded with approximately 10^6^ cells, which were incubated for 24 h at 37 °C in 5% CO_2_. The cells were then cultured in media supplemented with 400 nmol/L ethanol (treated control), 1000 nmol/L ethanol (treated control), 400 nmol/L cholecalciferol (treatment drug), or 1000 nmol/L cholecalciferol (treatment drug). Untreated cells served as control.

Dose standardization of cholecalciferol is mentioned in Supplementary Material. After the treatment, cells were kept at 37 °C in a 5% CO_2_ incubator for 24 h (Day 1), 48 h (Day 2), and 72 h (Day 3) and extracted by trypsin-EDTA on the respective days. The cell suspension thus extracted was aspirated and centrifuged at 1000× *g* for 5 min, and the cell pellet was stored at −80 °C until further analysis. The supernatant from HAECs were collected at respective time points and stored at −80 °C for nitrite/nitrate measurements. Each experiment was repeated three times in a duplicate set for both HAECs and HASMCs. mRNA and protein expression levels of the following markers were assessed: (1) Endothelial cell function markers: endothelial nitric oxide synthase (eNOS), mitogen-activated protein kinase 38 (p38 Map kinase), protein kinase B (Akt), and nicotinamide adenine dinucleotide phosphate oxidase (NADPH oxidase) by real-time polymerase chain reaction (RT-PCR) and Western blot, respectively. Cell supernatant nitrite concentrations in supernatants were measured using colorimetric assay. (2) Vascular smooth muscle cell function markers: α-smooth muscle actin (α-SMA), smooth muscle calponin (SM-Calponin), smooth muscle myosin heavy chain (SM-MHC), and calcium-sensing receptor (CaSR). (3) mRNA analysis of vitamin D-responsive genes: VDR and CYP27B1 (1-α hydroxylase; cytochrome P450, family 27, subfamily B, member 1).

### 4.4. mRNA Expression Analysis

RT-PCR was utilized to measure the expression levels of mRNA transcripts for endothelial and vascular smooth muscle cell function markers, along with those for, VDR and CYP27B1. Moreover, 18S rRNA was used as an endogenous control (housekeeping gene). Total RNA was extracted using the RNeasy^®^ Mini Kit (Qiagen, Hilden, Germany, Cat. No. 74104) and quantified using a spectrophotometer (Bio-photometer, Eppendorf, Germany). cDNA was prepared from 500 ng RNA using the First-Strand cDNA synthesis Kit (Invitrogen, Thermo-fisher Scientific, Waltham, MA, USA, Cat. No. 18091050) as per the manufacturer’s instructions. The TaqMan expression assay (ABI Biosystem, Foster City, CA, USA) was carried out on a real-time PCR machine (ABI Biosystem Prism 7500, ABI Biosystem, Foster City, CA, USA). A 20 μl reaction was prepared by mixing 10 μL of TaqMan^TM^ Fast Advanced Master Mix (Applied Biosystems, Thermofisher Scientific, Waltham, MA, USA, Cat. No. 4444557), 1 μL of TaqMan assay probe (Applied Biosystems, Thermofisher Scientific, Assay ID and amplicon length of probes as mentioned in Table S2), and 1 μL of cDNA template. The reaction mixture was incubated at 50 °C for 2 min, followed by polymerase activation at 95 °C for 20 s and 40 PCR cycles of denaturation at 95 °C for 3 s and annealing/extension at 60 °C for 30 s. The relative expression was calculated using the 2^−ΔΔCt^ method.

### 4.5. Protein Expression Analysis

A Western blot was performed on cell lysates of cultured HAECs and HASMCs to analyze protein expression. Total protein was extracted from stored experimental (treated and untreated) cell lysates using radioimmunoprecipitation assay lysis buffer (HiMedia Laboratories Pvt. Ltd., Thane, Maharastra, India, Cat. No. TCL131). The Bicinchoninic acid Protein Assay Kit (Sigma Aldrich, St. Louis, MO, USA, Cat. No. BCA1 and B9643) was used for protein estimation. Cell lysates were first subjected to sodium dodecyl sulfate–polyacrylamide gel electrophoresis, and then the resolved protein bands were transferred to polyvinylidene difluoride membranes (Thermo-fisher Scientific, Waltham, MA, USA, Cat. No. 88518), which were subsequently blotted using different primary antibodies against the respective protein markers. Furthermore, 50 μg of protein from each sample was loaded after mixing with 2× concentrated Laemmli buffer (HiMedia Laboratories Pvt. Ltd., Thane, Maharastra, India, Cat. No. ML021) in a 5:1 ratio. A 6–12% resolving gel followed by a 5% stacking gel was used. The loaded gel was subjected to electrophoresis for 2 h at 80 volts in the resolving buffer, followed by electrotransfer to a polyvinylidene difluoride membrane in a transfer buffer. After an overnight blocking with 3% bovine serum albumin (HiMedia Laboratories Pvt. Ltd., Thane, Maharastra, India, Cat. No. MB083), membranes were washed three times with Tris-buffered saline containing 0.1% Tween 20 (TBST) on a shaker for 10 min and incubated for 2 h with primary antibody (Table S3) against the respective endothelial and smooth muscle cell functional markers. After washing (three times with TBST), the membranes were incubated with the corresponding secondary antibody (Table S3) for 2 h. Furthermore, β-actin was used as an endogenous control. Bands were visualized by enhanced chemiluminescence on a chemi-doc instrument (Azure C400^®^, Azure Biosystems, CA 94568, USA). The band intensities were used to quantify proteins using ImageJ software version 1.54 (NIH, Bethesda, MD, USA). Protein expression is represented as a fold change in band intensities compared to the control (untreated) cells.

### 4.6. Measurement of Nitrite Concentration in HAEC Cell Supernatants

Nitrite concentrations were measured in supernatants of HAECs stored at −80 °C using the Total Nitric oxide and Nitrate/Nitrite assay (R&D Systems^®^, Minneapolis, MN, USA, Cat. No. KGE001). Optical density was determined at 540 nm using an ELISA Reader (BioTek^®^ PowerWave XS, Winooski, VT, USA). Concentrations of samples were obtained by equation of the best fit curve using automated software Gen5™ version 1.08 (BioTek Instruments, Winooski, VT, USA).

### 4.7. Statistical Analysis

Data were expressed as mean ± SD or median (IQR) for continuous variables and number and percentage for categorical variables, as appropriate. A *p*-value < 0.05 was considered significant. A paired sample *t*-test (Wilcoxon signed-rank test) was used for between-groups comparisons. Statistical Package for Social Sciences (SPSS Inc., Chicago, IL, USA, version 23.0) and GraphPad Prism 9.0 (San Diego, CA, USA) were used for analysis.

## Figures and Tables

**Table 1:** T-cell phenotype alterations upon the in vitro treatment with 400 or 1000 nmol/L cholecalciferol (*N* = 20).

T-Cell Subpopulation	T-Cell Marker	Untreated (Cell Percentage)	400 nmol/L Cholecalciferol (Cell Percentage)	*p*-Value	1000 nmol/L Cholecalciferol (Cell Percentage)	*p*-Value
TH1 cells	CD3^+^CD4^+^CXCR3^+^	28.9 ± 18.8	23.1 ± 14.1	0.42	23.5 ± 15.9	0.55
	CD3^+^CD4^+^Tbet^+^	26.6 ± 8.6	23.8 ± 9.6	0.31	25.0 ± 14.2	0.72
	CD3^+^CD4^+^IFNγ^+^	28.2 ± 7.7	20.7 ± 11.7	0.04	19.5 ± 12.5	0.02
TH2 cells	CD3^+^CD4^+^IL4^+^	31.8 ± 8.6	30.1 ± 11.9	0.38	31.9 ± 9.7	0.78
	CD3^+^CD4^+^STAT6^+^	17.1 ± 4.8	23.6 ± 11.3	0.02	25.0 ± 8.3	0.002
	CD3^+^CD4^+^GATA3^+^	27.7 ± 9.9	22.3 ± 5.7	0.07	23.3 ± 6.4	0.18
	CD3^+^CD4^+^CCR4^+^CCR6^-^	18.3 ± 5.4	24.1 ± 7.0	0.02	26.0 ± 9.8	0.009
TH17 cells	CD3^+^CD4^+^IL17A^+^	7.3 ± 5.4	4.5 ± 3.5	0.06	4.2 ± 2.8	0.06
	CD3^+^CD4^+^RORγt^+^	7.9 ± 7.0	4.1 ± 4.1	0.13	3.9 ± 3.8	0.07
	CD3^+^CD4^+^CCR4^+^CCR6^+^	15.4 ± 9.9	8.2 ± 8.3	0.02	10.0 ± 8.3	0.14
Treg cells	CD3^+^CD4^+^CD25^+^	14.8 ± 4.1	13.6 ± 6.2	0.53	14.0 ± 8.0	0.41
	CD3^+^CD4^+^CD25^+^CD127^low^FOXP3^+^	3.00 ± 1.2	3.3 ± 2.4	0.88	3.1 ± 1.7	0.79

Data presented as mean ± standard deviation. TH: T helper cells, Treg: T regulatory cells.

## Data Availability

The original contributions presented in this study are included in the article/Supplementary Material. Further inquiries can be directed to the corresponding author.

## Supplementary Materials

The following supporting information can be downloaded at: https://www.mdpi.com/article/10.3390/ijms26093967/s1.
