# Supplementary material for: In Vitro Study of Vitamin D Effects on Immune, Endothelial, and Vascular Smooth Muscle Cells in Chronic Kidney Disease
Source: Int J Mol Sci. 2025 Apr 23;26(9):3967. doi: 10.3390/ijms26093967 (PMC12071356; doi:10.3390/ijms26093967)
Supplement: Supplementary file 1 [file ijms-26-03967-s001.zip › ijms-3529940-supplementary.pdf]

Supplementary Methods

In-vitro **study of effect of cholecalciferol on immune cells, endothelial cells and vascular smooth muscle cells in CKD**

Kajal Kamboj[1]MSc, PhD; Vivek Kumar[1]MD, DM; Ashok Kumar Yadav[2]MSc, PhD;

Departments of [1]Nephrology, Postgraduate Institute of Medical Education and Research, Chandigarh, India;

[2]Experimental Medicine and Biotechnology, Postgraduate Institute of Medical Education and Research, Chandigarh, India;

Supplementary Method

Study population for T cell phenotyping

Subjects with pre-dialysis CKD attending the out-patient clinic of Department of Nephrology at Postgraduate Institute of Medical Education and Research (PGIMER), Chandigarh, India which were enrolled in ongoing trial were selected. 20 CKD subjects were selected with following inclusion and exclusion criteria: CKD patients between the ages 18-75 years, eGFR ($CKD-EPIcr_{2009}$) 15-60 $ml/min/1.73m^2$, serum 25(OH)D levels <20 ng/ml, clinically stable for at least 3 months were enrolled. CKD patients receiving immunosuppressive therapy, diagnosis of diabetes mellitus, chronic liver disease, primary hyperparathyroidism, sarcoidosis or malignancy, history of having been treated for hypercalcemia due to any cause, anticipated to need long-term renal replacement therapy within 6 months, poor functional status as judged by the physician, life expectancy <1 year as judged by the treating physician, pregnancy in case of females, smoking history, blood hemoglobin <8 g/dl, serum calcium >9.5 mg/dl, history of having received any type of organ transplantation, patients already on or having received vitamin D supplementation in last 30 days or participating in another interventional trial, history of allergy to interventional drugs (cholecalciferol) were excluded. All patients provided written informed consent before enrolment. The study protocol was approved by Institute's Ethics Committee (IEC) of PGIMER, Chandigarh, India. (No: NK/6731/PhD/930 dated 01/12/2020). The study was registered in clinical trial registry of India (CTRI/2019/10/021494). All participants provided informed consent before enrolment in the study. Enrolled patients were given two high dose (300000 IU each) of cholecalciferol at baseline and at 8 week. T cell phenotypes were analysed at baseline and at 16 weeks.

Standardisation of treatment with cholecalciferol

Stock cholecalciferol solution was prepared by dissolving 19.2 mg of cholecalciferol (Sigma-Aldrich, Cat no. C1357) powder in 50 ml ethanol in dark. Different concentrations of cholecalciferol ranging from 100-1000nmol/l (i.e. 100, 200, 400, 600, 800, 1000 nmol/l) were prepared in respective culture media by serial dilutions. Ethanol was taken as treated control (vehicle). Similar ethanol concentration were prepared by dissolving ethanol in Dulbecco's Phosphate Buffer Saline (DPBS, Cat No. D8662) and different concentrations were prepared in

culture media by serial dilutions. Human aortic smooth muscle cells (HASMCs) were used for dosage standardization. 50,000 cells per well were seeded in a 6 well plate followed by 24 hours incubation in 5% CO_2 at 37°C temperature. Experimental compound cholecalciferol and ethanol (control) ranging from 100-1000nmol/l were added in respective labelled wells. 3-(4,5- Dimethylthiazol-2-yl)-2,5-diphenyltetrazolium bromide for (MTT) (Sigma Aldrich, Cat No. 475989) assay was performed to examine the cellular viability, proliferation and dosage toxicity at day 1 (24 hours after treatment), day 2 (48 hours after treatment), day 3 (72 hours after treatment). As all the treatment doses were non-toxic for the cells, 400 nmol/l and 1000 nmol/l were chosen for further experiments.

Table S1: Various T cell phenotypes markers and labelled antibodies

T Cell phenotype	Surface Markers (conjugated dyes)	Intracellular Marker (conjugated dyes)	Transcription Factor (conjugated dyes)
Panel 1: TH1 Cells	CD3(FITC),CD4(BV510), CXCR3(PE)	IFNγ(APC)	T-bet (BV421)
Panel 2: TH2 Cells	CD3(FITC),CD4(BV510), CCR4+(PE), CCR6-(BB700)	IL4(BV421)	STAT6 (AF647), GATA3 (PE-Cy7)
Panel 3: TH17 Cells	CD3(FITC), CD4(BV510), CCR4+(PE), CCR6+(BB700)	IL17A(BV421)	RORγt (APC)
Panel 4: Treg Cells	CD3(FITC), CD4(BV510), CD25(BV421), CD127^{low}(AF647),CD45RA-(PE)	FOXP3(BB700)	FOXP3(BB700)

AF647: Alexa Fluor 647, APC: Allophycocyanin (APC), BV421: Brilliant Violet™ 421, BV510: Brilliant Violet™ 510, BB700: Brilliant™ Blue 700, FITC: fluorescein isothiocyanate, FOXP3: forkhead box protein 3, GATA3: GATA binding protein 3, IFN-γ: Interferon-γ, IL-4: Interleukin-4, IL-17A: Interleukin-17A, T-bet: T box transcription factor, TH1: T helper 1 cell population, TH2: T helper 2 cell population, TH17: T helper 17 cell population, Treg: T regulatory cell population, PE: phycoerythrin, , PE-Cy7: phycoerythrin-cyanine7, RORγt: retinoic-acid-receptor-related orphan nuclear receptor gamma transcription factor, STAT6: signal transducer and activator of transcription 6

Table S2: Assay ID and amplicon length of probes of qRT-PCR experiments

Gene Name	NCBI reference sequence ID	ABI assay ID	Amplicon length (BP)
18s	18s_consensus.0	Hs03003631_g1	69
eNOS	NM_000603.4	Hs01574659_m1	107
p38 Map kinase	NM_001315.2	Hs01051152_m1	91
protein kinase B(Akt)	NM_001014431.1	Hs00178289_m1	66
NADPH oxidase	NM_001143836.2	Hs00418356_m1	109
α-SMA	NM_001141945.2	Hs05005341_m1	83
SM-MHC	NM_001040113.1	Hs00975796_m1	53
SM-Calponin	NM_001299.5	Hs00154543_m1	93
VDR	NM_000376.2	Hs00172113_m1	62
CYP27B1	NM_000785.3	Hs00168017_m1	60

Table S3: Antibodies and their dilution used for western blot.

Antibody Name	Cat. No.	Dilution used (Ab: PBST)
P38MAPK (monoclonal mouse ab)	66234-1-Ig (Proteintech®)	1:1000
PKB(AKT) (monoclonal mouse ab)	60203-2-Ig (Proteintech®)	1:2000
NOX4 (rabbit polyclonal ab)	14347-1-AP (Proteintech®)	1:2000
eNOS (mouse monoclonal ab)	MA5-15559 (Invitrogen)	1:2000
α-SMA (mouse monoclonal ab)	14395-1-AP (Invitrogen)	1:2000
SM-Calponin (mouse monoclonal ab)	66540-1-Ig (Proteintech®)	1:2000
SM-MHC (mouse monoclonal ab)	60222-1-Ig (Proteintech®)	1:1000
CaSR (rabbit polyclonal ab)	19125-1-AP (Proteintech®)	1:2000
Beta Actin (mouse monoclonal ab)	66009-1-Ig (Proteintech®)	1:5000
Secondary ab Goat Anti-Rabbit	SA00001-2 (Proteintech®)	1:5000
Secondary ab Goat Anti-Rat	SA00001-15 (Proteintech®)	1:5000
Secondary ab Rabbit Anti-Goat	SA00001-4 (Proteintech®)	1:5000
Secondary ab Goat Anti-Mouse	SA00001-1 (Proteintech®)	1:5000

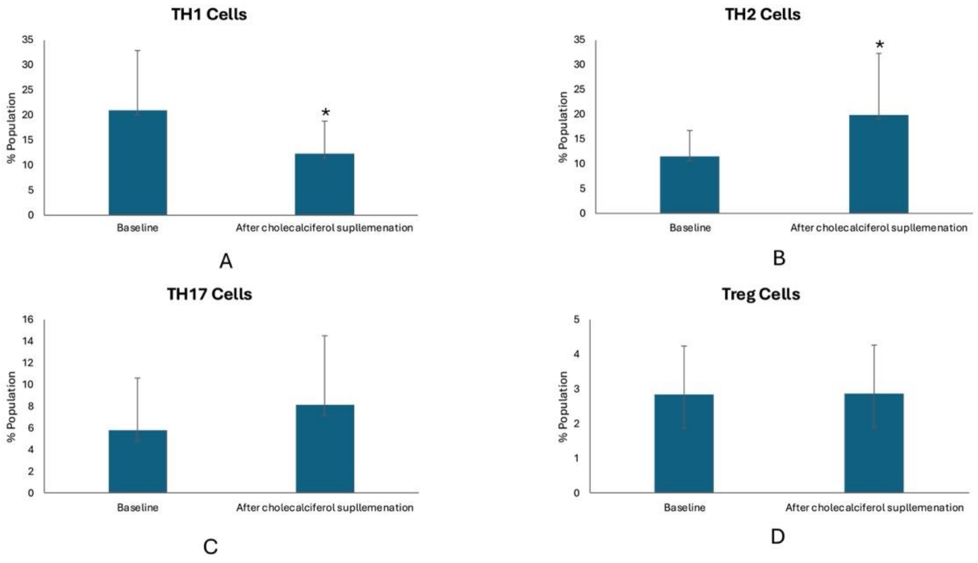

Figure S1: Representative bar graph of T cell subpopulations in CKD patients at baseline and 16 weeks follow up (after cholecalciferol supplementation) (A) TH1 $CD3^+CD4^+IFN\gamma^+$ population (B) TH2 $CD3^+CD4^+STAT6^+$ (C) TH17 $CD3^+CD4^+IL17A^+$ (D) Treg $CD3^+CD4^+CD25^+CD127^{low}FOXP3^+$ population. *$p<0.05$. p- values were obtained using Wilcoxon signed-rank test.

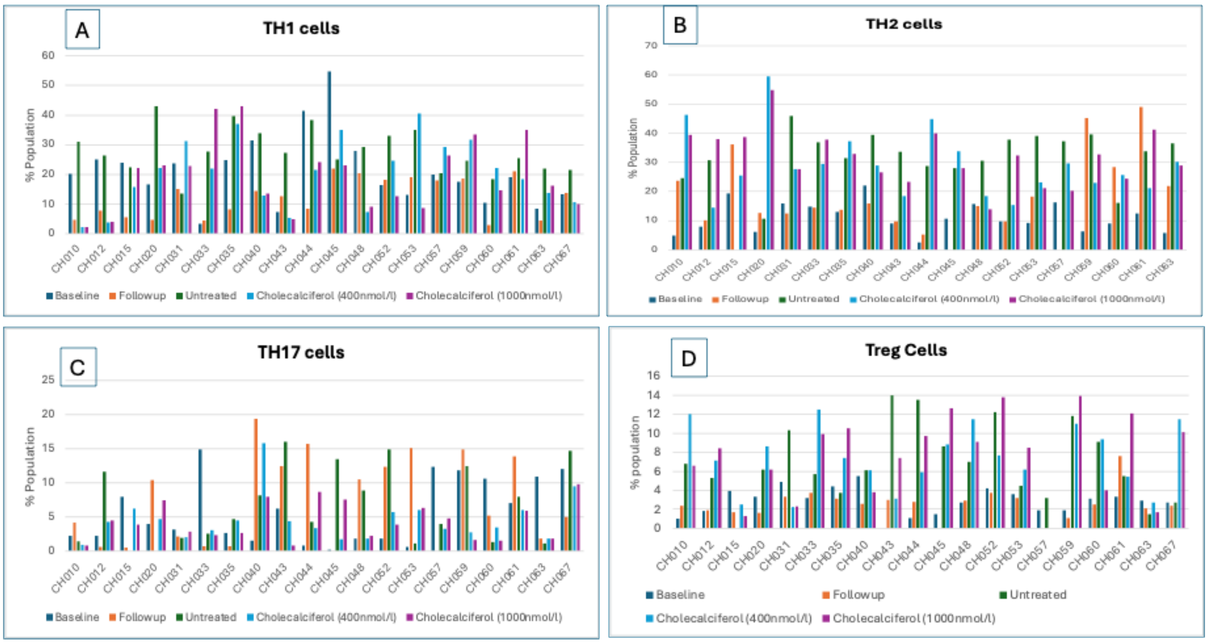

Figure S2: Individual level data representing various T cell subpopulations in CKD subjects at baseline, follow up (16 weeks after cholecalciferol supplementation) and their PBMCs: untreated, 400 nmol/l cholecalciferol treatment, 1000 nmol/l cholecalciferol treatment. (A) TH1 $CD3^{+}CD4^{+}IFN\gamma^{+}$ (B) TH2 represents $CD3^{+}CD4^{+}STAT6^{+}$ (C) TH17 represents $CD3^{+}CD4^{+}IL17A^{+}$ (D) Treg $CD3^{+}CD4^{+}CD25^{+}CD127^{low}FOXP3^{+}$ cell population.

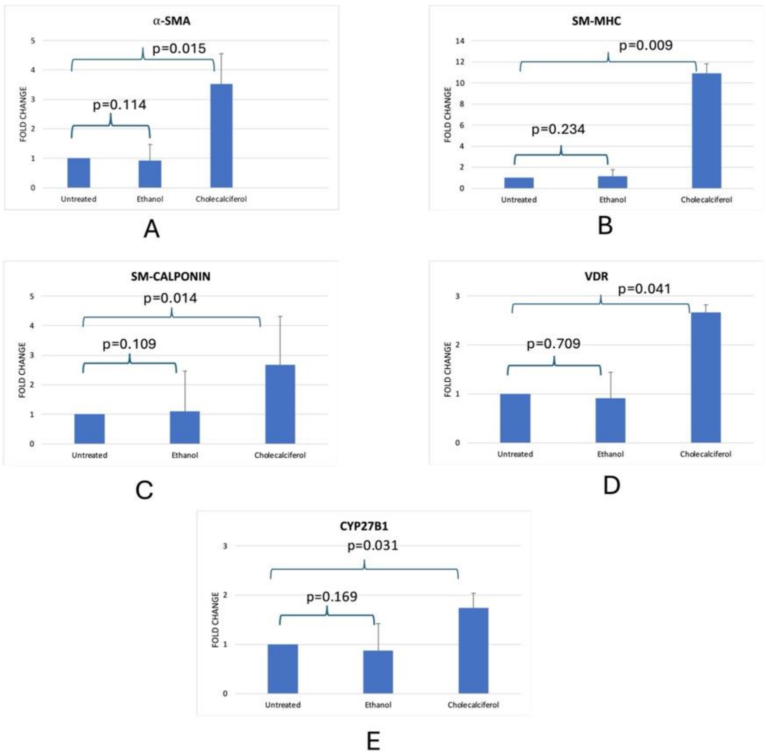

Figure S3: mRNA expression of markers of HASMCs in untreated control, treated control (ethanol) and treatment drug (cholecalciferol). p-value is for comparison between untreated versus ethanol (treatment control) and untreated versus cholecalciferol treatment. α-SMA: α-smooth muscle actin, SM-MHC: smooth muscle myosin heavy chain, SM-Calponin: smooth muscle calponin, VDR: vitamin D receptor, CYP27B1: cytochrome P450 family 27 subfamily B member 1.

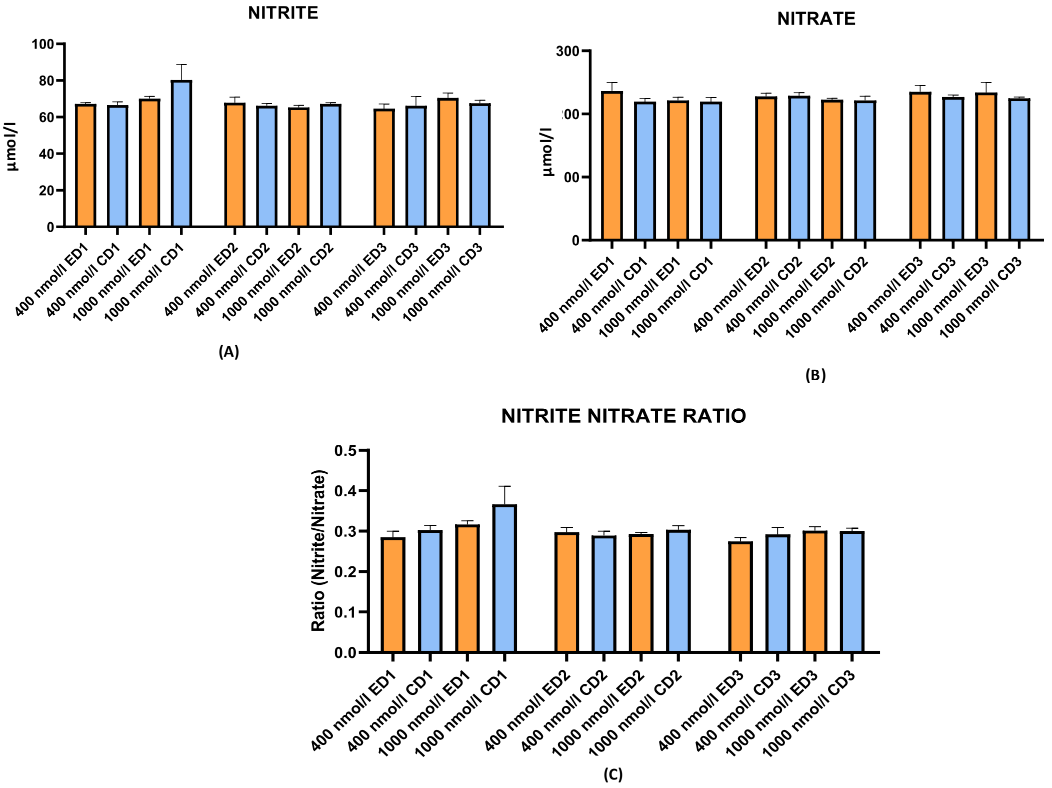

Figure S4: Graphical representation of concentrations of nitrite and nitrate in human aortic endothelial cells (HAECs) supernatants as analysed by nitrite-nitrate assay after treatment with either 400 or 1000 nmol/l of ethanol or cholecalciferol. (A) Levels of nitrite (B) Levels of nitrate (C) Levels of nitrate and nitrate ratio. ED1- Ethanol Day 1, CD1-Cholecalciferol Day 1, ED2- Ethanol Day 2, CD2- Cholecalciferol Day 2, ED3- Ethanol Day 3, CD3- Cholecalciferol Day 3
